# Effect of different fixed lingual retainers on tooth mobility: prospective clinical study

**DOI:** 10.2340/aos.v84.44808

**Published:** 2025-12-10

**Authors:** Eyüp Burak Küçük, Özge Çelik Güler, Osman Fatih Arpağ, Hatice Kübra Eren

**Affiliations:** a Department of Orthodontics, Faculty of Dentistry, Hatay Mustafa Kemal University, Hatay, Türkiye; b Private Practice, İdadent Dental Clinic, Çanakkale, Türkiye; c Department of Periodontology, Faculty of Dentistry, Hatay Mustafa Kemal University, Hatay, Türkiye

**Keywords:** Retention and stability, tooth mobility, Periotest

## Abstract

**Objective:**

The aim of this study was to compare the effects of different fixed lingual retainers (LRs) on tooth mobility after orthodontic treatment at the 10-month follow-up.

**Methods:**

72 patients were allocated to three different LR groups (Ortho FlexTech^®^ chain, Bond-A-Braid^®^, Penta-One^®^). Additionally, 20 non-treated people were included as a control group. The mobility of six mandibular anterior teeth was measured using a Periotest^®^ device at each time points (before application of LR [T_0_], immediately after application of LR [T_1_] and 10 months after [T_2_] the application of the LR), and at only one time (T_0_) for the control group.

**Results:**

The mobility value was significantly lower at T_1_ and T_2_ compared to that at T_0_ (*P* < 0.05); yet, there were no differences at T_1_ and T_2_ in all LR groups (*P* > 0.05). No significant differences in mobility between the T_0_-T_1_, T_0_-T_2_, and T_1_-T_2_ time points were observed across the LR groups (*P* > 0.05). The mobility of teeth 33 and 43 decreased over time across all study groups (*P* < 0.05).

**Conclusions:**

Mobility decreased immediately after LR application, but did not change during the 10-month follow-up in the treatment groups. The observed decrease in mobility of canines over time, in contrast to other teeth, suggests a relationship between root size and mobility.

**Trial registration:**

The current clinical trial was recorded at clinicaltrials.gov, NCT06284499, 08/02/2024, retrospectively registered.

## Introduction

After orthodontic treatment is completed, any adverse change in the position of the teeth associated with a malocclusion correction is referred as relapse. Several factors influence the incidence of relapse, including the elastic property of gingival fibers, lip/tongue pressure, and jaw growth during the retention period [1]. Changes in tooth position are also observed to occur due to normal age-related factors [2]. Thus, action is often taken during the subsequent retention period to ensure that the teeth remain in their new positions after orthodontic treatment [3]. Sufficient periodontal tissue must form around the root for successful tooth stability in a new position, and the soft tissues around the tooth must also adapt to the new tooth position.

A lingual retainer (LR) can be used during the retention period to maintain the ideal alignment of teeth after orthodontic treatment and prevent relapse [4]. However, LRs must both prevent relapse and allow physiological tooth movements [5]. To date, various types of wire have been used for retention, among which 6-strand coaxial wire [5, 6], rectangular 8-strand braided wire [6], flat braided chain wire [7], and computer-aided design and computer-aided manufacturing (CAD/CAM) customized retainers [8] are so far the most used.

In fact, low-level tooth mobility is well-known phenomenon during the retention phase. Different LRs are also well understood to provide different levels of stability; therefore, it is important to assess the extent to which LRs provide the best stability. Even though there have been several methods to measure this mobility, there is still a lack of standardization, which might lead to slight measurement uncertainty or intra clinical changes. Among them, the Miller mobility classification [9] is widely used in clinical settings to assess tooth mobility. Although its utilization ensures rapid decision-making, it is a relatively subjective method. In contrast, a Periotest ® device can be used to objectively evaluate tooth mobility in an easy manner without the need for any contact [10].

To the best of our knowledge, no studies have compared the immediate and 10-month follow-up changes in lower anterior tooth mobility after LR application. The null hypothesis of this study was that there were no differences between the LR wires according to the mobility of lower anterior teeth, which was measured with Periotest^®^ device. Hence, this study was designed to investigate changes in tooth mobility during the retention phase after orthodontic treatment in patients with three different types of LR.

## Materials and methods

### Ethical approval and sample size calculation

Ethical approval was granted by the Human Ethics Committe of the University of Hatay Mustafa Kemal with the number 2020/21. The participants were informed about the purpose of the study, and a signed informed consent was obtained from each. All methods were carried out in compliance with the relevant guidelines and regulations.

In this prospectively planned study, a pilot study was conducted with 57 patients, and a post hoc power analysis was performed using the mobility data obtained from these patients (G power program version 3.1.9.7, Franz Faul, Kiel, Germany). This analysis, calculated based on the mobility means among groups, indicated that when the total number of patients included in the study reached 92, the effect size was *f* = 0.42, and the study power was 95% [10]. Accordingly, a total of 92 patients were evaluated: 72 patients with a fixed LR as treatment group; and 20 people who did not receive any orthodontic treatment as control group.

### Patient selection

The inclusion criteria were as follows: patients who had applied a fixed LR (bonded to all six anterior teeth) on the mandibular teeth from canine to canine; successful completion of fixed orthodontic treatment with Class I occlusion and optimum oral hygiene; a treatment period with fixed appliance of at least 12 months; no missing teeth, restorations, apical root surgery, or morphological crown anomalies; and no radiological alveolar bone loss in the mandibular inter-canine region. Patients with periodontal status, such as gingival recession; parafunctional habits (e.g. clenching and grinding); and any radiological pathology around the relevant teeth were excluded from the study. The control group was subjected to the same inclusion criteria as the study group.

### Study groups

The subjects (*n* = 72) were divided into three treatment groups: Group 1 (*n* = 21), 0.038×0.016-in stainless steel Ortho FlexTech^®^ chain retainer (Reliance Orthodontic Products, Itasca, IL, USA); Group 2 (*n* = 23), 0.010×0.026-in 8-braided dead soft Bond- A-Braid^®^ retainer (Reliance Orthodontic Products, Itasca, IL, USA); and Group 3 (*n* = 28), 0.0215-in 5-stranded stainless steel Penta-One^®^ wire (Masel Orthodontics, Carlsbad, CA, USA). Since the control group (Group 4) consist of non-orthodontic-treated individuals (n=20), only one set mobility measurements was conducted for these objects.

### Fixed orthodontic treatment

The fixed orthodontic treatments, including extraction and non-extraction cases, were carried out at a university orthodontic department. Patients were treated with MBT bracket system (0.018-in slot size, Gemini, 3M Unitek, Monrovia, CA, USA), and nickel-titanium arch wires (sized between 0.012-in, 0.017-in×0.025-in). To provide ideal inclination and angulation of teeth, 0.016-in×0.022-in and 0.017-in×0.025-in stainless steel wires were used. After achieving the treatment objectives, fixed orthodontic appliances were removed and the phase of retention was initiated.

### LR wire preparation and application

**Figure 1:**
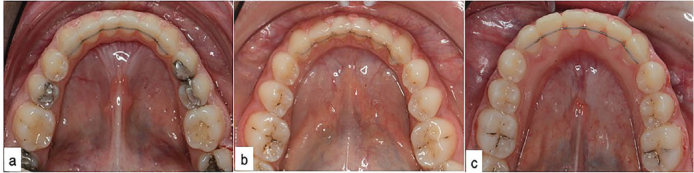
The application of Ortho FlexTech® chain (a), Bond-A-Braid® (b), and Penta-One® (c) LR wires.

LRs were fitted on six anterior teeth (33–43) on the mandible as follows:

In Group 1, Orthoflex retainer chain (Figure 1a) was applied directly at the chairside and shaped passively to the lingual tooth surfaces.

In Groups 2 and 3, before debonding session, a plaster model of mandibular arch was obtained. An experienced technician shaped the selected retention wire to passively fit on the lingual surface of the relevant teeth on this model. A silicone carrier tray was prepared for all pre-shaped LR wires to ensure the wire was positioned correctly.

A prophylaxis with ultrasonic dental scaler (DTE D5 LED, Woodpecker, China) was performed for patients in need prior to LR application. The lingual surfaces of canines and incisors were etched (37% phosphoric acid gel, Reliance Orthodontic Products) for 30 s, rinsed with water, and dried sufficiently with air. A thin coat of Transbond XT Primer (3M Unitek, Monrovia, Calif) was then applied to the lingual enamel surfaces, thinned with gentle air and, polymerized for 10 s using an LED light device (Woodpecker Medical Instrument Co., Guilin, China). The LR wire, which was pre-positioned on the plaster model and fixed with a silicone transfer tray, was placed in the correct position in the mouth, and Transbond LR adhesive (3M Unitek, Monrovia, Calif) was applied over the wire. Care was taken to ensure that the adhesive did not overflow into the proximal areas of the teeth and sufficiently covered the wire with an adequate layer. Adhesive was polymerized for 30 s for each tooth. After removing the silicon carrier, adhesive was applied to the tooth under the carrier (Figure 1b-c). All of the retainers were applied by the same orthodontist (EBK).

### Measuring tooth mobility

The mobility of the six mandibular anterior teeth (33–43) was measured using a calibrated Periotest^®^ device (Medizintechnik Gulden, Modautal, Germany) with a standard protocol at the T_0_ (after the removal of fixed appliances and adhesive remnants, before LR application); T_1_ (immediately after the LR application) and; T_2_ (10 months after the LR application) timepoints.

**Figure 2:**
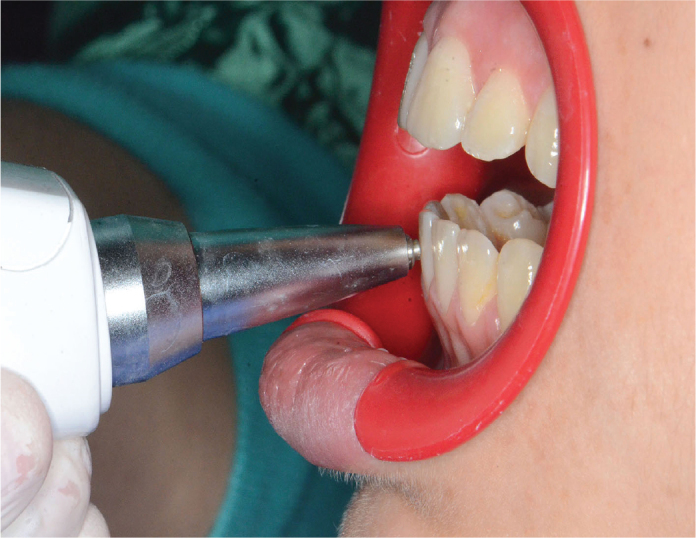
Measurement of mobility using Periotest® device.

The measurements were carried out according to the manufacturer’s instructions following the method of Berthold et al. [10] by a periodontist (OFA), who held the Periotest^®^ device while the patient was sitting in the dental unit in an upright position. The head of the instrument was placed at a right angle to the buccal surfaces of the clinical crowns for the tooth being assessed in the lower anterior segment and maintained a distance between 0.5 and 2 mm from the tooth (Figure 2). During the measurements, an audio signal confirmed the proper positioning of the device. The mobility was determined by taking three repeated measurements, as mentioned previously [11], from each tooth and calculating the mean of these measurements. In this study, all the recorded mobility values were within the physiological mobility limit [12].

### Statistical analysis

Groups that were found to be normally distributed using the Shapiro-Wilk test were compared using one-way ANOVA test, and post-op evaluations were performed using post hoc LSD test. Repeated ANOVA with Bonferroni post hoc tests were used to analyze features with normal distribution at different time points. Intergroup comparisons of categorical variables were conducted using the Pearson χ^2^ test. The reliability of the measurements taken at different times was evaluated with the one-way random effects model intraclass correlation coefficient (ICC). Descriptive statistics are shown as means and standard deviations for numerical variables, and as numbers and percentages for categorical variables. The data were analyzed using SPSS software (version 23.0; IBM, Armonk, NY, USA), and a *P-*value < 0.05 was considered significant.

## Results

The ICC for all measurements was 0.948–0.989, which indicated good agreement (ICC > 0.810) among all measurements. Of the 72 patients studied, for which the general characteristics are shown in Table 1, all completed the 10-month follow-up successfully. There were no significant differences between the treatment groups in terms of gender and treatment time (*P* > 0.05). However, the mean age in the control group was significantly higher than in the treatment groups (*P* < 0.05).

**Table 1:** General characteristics of the participants.

Group	1	2	3	4	*P*
**Gender**					
Male	8 (38.1)	10 (43.5)	11 (39.3)	10 (50)	0.860
Female	13 (61.9)	13 (56.5)	17 (60.7)	10 (50)	
**Age** (mean ± SD)	^a^17.33 ± 2.65	^a^19.04 ± 3.01	^a^17.32 ± 2.50	^b^21.25 ± 5.25	0.001
**Treatment Time** (mean ± SD)	30.81 ± 10.13	32.61 ± 9.24	27.35 ± 9.28	-	0.141

SD: standard deviation.

Within each row, different letters in superscript indicate differences (*P* < 0.05) according to the LSD test.

In this study, the six teeth between the mandibular canines were individually measured at three different times for mobility, and the mean values of all six teeth in the groups, as well as the individual mean mobility of the teeth within the group, were analyzed. When the mean tooth mobility was compared within and among the groups (Table 2), it was found that the mobility at T_1_ and T_2_ was significantly lower than at T_0_ (*P* < 0.05); however, there was no difference between T_1_ and T_2_ (*P* > 0.05) in all treatment groups. The mean mobility values at T_0_, T_1_, and T_2_ time points do not differ significantly between the treatment groups; however, these values are significantly higher than the control group at T_0_, while they are lower than the control group at T_1_ and T_2_ times. As shown in Table 3, no significant differences in mobility between the two different time points (T_0_-T_1_, T_0_-T_2_, and T_1_-T_2_) were also observed across the LR groups (*P* > 0.05).

**Table 2:** Comparison of mean tooth mobility within and between groups.

Group	T_0_ Mean ± SD	T_1_ Mean ± SD	T_2_ Mean ± SD	*P§*
**1**	^b^5.01 ± 3.03^*^	0.13 ± 1.93^†^	0.14 ± 1.40^†^	< 0.001
**2**	^b^5.91 ± 4.79^*^	0.24 ± 2.38^†^	0.90 ± 2.89^†^	< 0.001
**3**	^b^4.84 ± 3.58^*^	0.85 ± 2.25^†^	0.96 ± 2.06^†^	< 0.001
**4**	^a^1.63 ± 1.65	ND	ND	-
*P* ^*^	0.001	0.089	0.169	-

* ANOVA test.

§ : Repeated measures ANOVA; SD: Standard Deviation, *P* < 0.05.

Within each column, different letters in superscript indicate differences (*P* < 0.05) according to the LSD test. Within each row, different symbol (*,^†^) in superscript indicate differences (*P* < 0.05) according to the Bonferroni corrected post hoc test. ND: Not determined owing to non-treatment procedure.

**Table 3:** Examination of tooth mobility changes (T_0_-T_1_, T_0_-T_2_ and T_1_-T_2_) between groups.

Group	*N*	T_0_–T_1_ Mean ± SD	T_0_–T_2_ Mean ± SD	T_1_–T_2_ Mean ± SD
**1**	21	4.88 ± 2.32	4.87 ± 2.33	−0.01 ± 1.33
**2**	23	5.66 ± 3.41	5.01 ± 3.77	−0.65 ± 1.98
**3**	28	3.98 ± 2.09	3.87 ± 2.55	−0.11 ± 1.60
**Total**	72	4.78 ± 2.70	4.52 ± 2.95	−0.25 ± 1.66
*P* ^*^		0.084	0.327	0.382

ANOVA test, SD: standard deviation.

In Tables 4 and 5, the mean mobility values of each individual tooth at each different time point (T_0_, T_1_, and T_2_) were given. The mobility of tooth 43 significantly decreased over time in Groups 1 and 2 (*P* < 0.05); however, it was not changed from T_1_ to T_2_ in Group 3 (*P* > 0.05; Table 4). The mobility of teeth 41 and 42 significantly decreased in Group 1 over time (*P* < 0.05); however, the mobility of these same teeth increased in Groups 2 and 3 from T_1_ to T_2_ (*P* < 0.05; Table 4).

**Table 4:** Post hoc test results in repeated measurements within the group for teeth of 41, 42, and 43.

Group	41				42				43			
	T_0_	T_1_	T_2_	*P§*	T_0_	T_1_	T_2_	*P§*	T_0_	T_1_	T_2_	*P§*
	Mean ± SD	Mean ± SD	Mean ± SD		Mean ± SD	Mean ± SD	Mean ± SD		Mean ± SD	Mean ± SD	Mean ± SD	
**1**	9.07 ± 4.42^‡^	1.82 ± 2.33^†^	1.64 ± 1.70^*^	**< 0.001**	5.50 ± 3.68^‡^	0.67 ± 2.22^†^	0.56 ± 1.99^*^	**< 0.001**	0.69 ± 2.06^‡^	−0.89 ± 2.34^†^	−1.43 ± 1.73^*^	**0.001**
**2**	10.50 ± 7.12^‡^	1.66 ± 2.75^*^	2.80 ± 3.92^†^	**< 0.001**	6.28 ± 5.70^‡^	0.21 ± 2.45^*^	1.13 ± 3.14^†^	**< 0.001**	1.02 ± 3.33^‡^	−1.12 ± 2.67^†^	−1.17 ± 2.60^*^	**0.001**
**3**	9.04 ± 5.89^‡^	2.52 ± 2.96^*^	3.42 ± 2.58^†^	**< 0.001**	5.51 ± 4.34^‡^	1.12 ± 2.30^*^	1.35 ± 2.45^†^	**< 0.001**	0.24 ± 2.17^†^	−1.25 ± 1.78^*^	−1.65 ± 2.14^*^	**0.001**
**4**	4.48 ± 2.61	ND	ND		1.73 ± 2.14	ND	ND		−1.30 ± 2.04	ND	ND	
*P* ^*^	**0.003**	0.488	0.109		**0.003**	0.384	0.563		**0.017**	0.858	0.741	

* : ANOVA test,

§ : Repeated measures ANOVA; SD: standard deviation.

Within each row, different symbol (^*^,^†^,^‡^) in superscript indicates differences (*P* < 0.05) according to the Bonferroni corrected post hoc test. ND: Not determined owing to non−treatment procedure.

**Table 5:** Post hoc test results in repeated measurements within the group for teeth of 31, 32, and 33.

Group	31				32				33			
	T_0_	T_1_	T_2_	*P§*	T_0_	T_1_	T_2_	*P§*	T_0_	T_1_	T_2_	*P§*
	Mean ± SD	Mean ± SD	Mean ± SD		Mean ± SD	Mean ± SD	Mean ± SD		Mean ± SD	Mean ± SD	Mean ± SD	
**1**	8.50 ± 5.03^†^	0.89 ± 2.24^*^	1.48 ± 1.90^*^	**<0.001**	5.95 ± 3.36^†^	0.02 ± 2.15^*^	0.35 ± 1.56^*^	**<0.001**	0.36 ± 2.48^‡^	−1.73 ± 2.09^†^	−1.74 ± 1.99^*^	**0.001**
**2**	10.84 ± 7.30^‡^	1.23 ± 3.14^*^	2.57 ± 3.71^†^	**<0.001**	5.88 ± 5.46^†^	0.47 ± 2.62^*^	1.03 ± 2.89^*^	**<0.001**	0.92 ± 2.94^‡^	−0.98 ± 2.61^†^	−0.98 ± 2.81^*^	**0.001**
**3**	8.68 ± 5.75^‡^	2.65 ± 2.61^*^	3.29 ± 2.68^†^	**<0.001**	5.33 ± 4.04^‡^	1.36 ± 2.79^†^	1.15 ± 1.99^*^	**<0.001**	0.24 ± 2.70^‡^	−1.27 ± 2.57^†^	−1.77 ± 2.09^*^	**0.001**
**4**	4.29 ± 1.96	ND	ND		2.31 ± 1.79	ND	ND		−1.73 ± 2.15	ND	ND	
*P* ^*^	**0.002**	0.055	0.101		**0.011**	0.182	0.430		**0.009**	0.594	0.422	

* : ANOVA test.

§ : Repeated measures ANOVA; SD: standard deviation, *P* < 0.05.

Within each row, different symbol (^*^,^†^,^‡^) in superscript indicates differences (*P* < 0.05) according to the Bonferroni corrected post hoc test. ND: Not determined owing to non-treatment procedure.

In all the groups, the mobility of tooth 33 significantly decreased over time (*P* < 0.05; Table 5). There was a significant decrease at T_1_ (*P* < 0.05), but no difference at T_2_ in Groups 1 and 2 for tooth 32 (*P* > 0.05; Table 5). There was a significant decrease in the mobility of tooth 32 in Group 3 across all the time points (*P* < 0.05; Table 5). The mobility of tooth 31 was found to be significantly lower at T_1_ when compared to T_0_ (*P* < 0.05) and found to be similar at T_1_ and T_2_ in Group 1 (*P* > 0.05; Table 5). In Groups 2 and 3, the mobility of tooth 31 was significantly lower at T_1_ when compared to T_0_ (*P* < 0.05), and it was found to be higher at T_2_ than at T_1_ (*P* < 0.05; Table 5).

## Discussion

It is important that the new tooth positions are maintained after lengthy and effective orthodontic treatment. Nowadays, patients are more demanding during the treatment process because they view content on social media platforms that influences their perceptions of potential outcomes and increases their awareness of the risk of relapse occurring [13]. The possibility that treated teeth may return to their original state is alarming for the patient and orthodontist alike, and treated teeth are very prone to relapse in the first weeks after debonding [13]. Therefore, in this study, we compared the mobility of the six lower anterior teeth, which were retained with different types of LRs, before and after retainer application and 10 months later. To our knowledge, no other study has examined tooth mobility with different wires 10 months after LR application. The results showed that the use of the tested retainer wires led to similar tooth mobility values after bonding and at the 10-month follow-up and that these changes were within physiological limits, so the null hypothesis was not rejected.

To date, many approaches have been applied to develop a precise method for assessing tooth mobility. For example, Mühlemann [14] utilized two different methods to examine the degree of mobility, O’Leary and Rudd [15] designed a device to measure tooth mobility in healthy individuals, and Burstone et al. [16] used laser holography to measure tooth mobility. These studies involved various tools and procedures that are difficult or impractical to perform routinely. In contrast, tooth mobility was evaluated using a Periotest^®^ device in this study, which is a simple and effective method [10].

Since cylindrical and flattened flexible stranded wires are most commonly used in LRs [17], here, we used flattened wire in two groups and cylindrical wire in one group. Previous studies [18–20] reported that the retainer wire thickness does not make a difference in the survival rates of mandibular fixed retainers. Engeler et al. [21] stated that effective torsional load transfer depends on the size, shape, and type of retainer wire and that plain and braided LRs have more predictable results in terms of torsional load transfer than multistrand LRs.

An earlier study conducted on tooth mobility reported that tooth mobility after orthodontic treatment was three times greater in the treatment groups with commonly used retainer wires than these in the control group [22]. The study by Kucera et al. [22] was, however, limited to the measurement after bonding of the retainer, for which we further evaluated the 10 month follow-up measurement. In our study, before LR application, the tooth mobility of the lower incisors was three times higher in the study groups than in the control group, and there was much less change in the mobility of the canines. In Kucera et al. [22] study, the incisors exhibited greater mobility after orthodontic treatment than in our study. Authors also found that the four tested types of wire significantly reduced tooth mobility, that the mobility was very close to the physiological mobility found in the control group, and that the type of wire used did not significantly impact the mobility. These authors [22] used four different wires (0.0175-in coaxial, 0.0150-in twist, 0.016-in×0.022-in rectangular flat dead, and 0.0215-in coaxial) and found that using 0.0150-in twist and 0.016-in×0.022-in rectangular flat dead wires reduced tooth mobility more than using the other two wires. Compared to the values reported by Kucera et al. [22], the mobility values recorded in our study before and after the application of the LR were considerably lower. In our study, the type of wire did not significantly affect mobility; however, the highest level and greatest change in mobility were recorded in the patients fitted with 0.010-in×0.026-in dead wire (Group 2).

The mobility of teeth 31, 41, and 42 decreased after the LR was applied, but it was found to have increased at the 10-month follow-up. Local anatomical factors or occlusal factors may have been responsible for this change within the physiological movement limits, which was not clinically significant. Additionally, the mobility value of teeth 33 and 43 decreased progressively from the T_0_ to the T_2_ time. This change, observed only in the canine teeth across all LR groups, suggests that the discrepancy in root size between canines and incisors, and consequently the increased root dimensions, could play a role in stability.

Hwang et al. [11] evaluated changes in tooth mobility at quarterly intervals for 2 years after orthodontic treatment using a Periotest^®^ device and found that tooth mobility decreased rapidly in the first 6 months and at a slower rate in the following 6 months. In the current study, tooth mobility decreased immediately after the LR was applied, and there was no significant difference in mobility among all the study groups 10 months later. The difference in the findings may be due to the fact that Hwang et al. [11] measured the mobility of the upper teeth without application of LR.

Tanaka et al. [23] measured the mobility of the central and lateral teeth in both arches using a Periotest^®^ device immediately before and after orthodontic treatment and after retention. Authors [23] used 0.015-in flexible multistrand wires in a lower canine-to-canine arrangement and found that tooth mobility increased after orthodontic treatment and decreased significantly over a 28-month retention period. We did not measure tooth mobility before orthodontic treatment. Nevertheless, our results aligned with theirs [23] in that (1) the tooth mobility values recorded in all three test groups after orthodontic treatment (T_0_) significantly differed from those of the control group, and (2) tooth mobility was significantly lower 10 months after LR application (T_2_) compared to immediately after orthodontic treatment (T_1_).

Watted et al. [12] used a Periotest^®^ device to evaluate and compare the mobility of the incisors 6 months after a canine–canine LR was bonded to two or six teeth. Their [12] participants consisted of patients fitted with either flexible multistrand wire (0.0175-in) that was bonded to all six anterior teeth, semi-round wire that was only bonded to the canines, or removable uni-maxillary appliances.

The mobility values recorded in our study are similar to those recorded by Watted et al. [12], in patients fitted with 0.0175-in wire bonded to six teeth; the mobility values for the incisors and canines are similar. Authors [12] suggested that the use of an LR may lead to atrophy of periodontal tissues by limiting physiological tooth movements. On this same issue, Baysal et al. [24] stated that LR wires must be flexible enough to allow a certain amount of physiological tooth movement to protect periodontal health and reduce the stress in the composite. Similar to previous studies [22, 23], in our study, the tooth movement observed immediately after and 10 months after LR application was within physiological limits.

The mobility values of this study were indicative of the stability created by applying the retainer wire to the teeth. In addition, comparing the tooth mobility values obtained at T_1_ and T_2_ allowed us to assess the 10-month effects of each type of wire. As a result, the data evaluated in this study were independent of the type of treatment or other variables. Additional studies must be conducted to confirm our findings and further evaluate the effects of retention wires on tooth mobility.

## Limitations

CAD/CAM retainers were not preferred in this study due to extra costs, external orders, and delays in delivery.^8^

## Conclusion

The LR wires assessed in this study were found to have similar effects on tooth mobility immediately after application and at the 10-month follow-up. All the tested types of LRs significantly reduced tooth mobility within physiological movement limits. Since different types of LRs establish similar stability, the choice of wire can be based on the clinician’s preferences and ease of application.

## Declarations

### Data availability

Data from this study will be shared with the corresponding author upon reasonable request.
